# Cultivable gut bacteria provide a pathway for adaptation of *Chrysolina herbacea* to *Mentha aquatica* volatiles

**DOI:** 10.1186/s12870-017-0986-6

**Published:** 2017-03-02

**Authors:** Graziano Pizzolante, Chiara Cordero, Salvatore M. Tredici, Davide Vergara, Paola Pontieri, Luigi Del Giudice, Andrea Capuzzo, Patrizia Rubiolo, Chidananda N. Kanchiswamy, Simon A. Zebelo, Carlo Bicchi, Massimo E. Maffei, Pietro Alifano

**Affiliations:** 1 Department of Biological and Environmental Sciences and Technologies, University of Salento, via Monteroni 165, 73100 Lecce, Italy; 2 Dipartimento di Scienza e Tecnologia del Farmaco, Università di Torino, Via Pietro Giuria n°9, 10125 Torino, Italy; 3 Dipartimento di Biologia, Sezione di Igiene, Institute of Biosciences and Bioresources-UOS Portici (IBBR-UOS Portici), CNR, Portici (NA) c/o, 80134 Naples, Italy; 4 Dipartimento di Scienze della Vita e Biologia dei Sistemi, Università di Torino, Via Quarello 15/A, 10135 Torino, Italy; 5 Research and Innovation Centre Genomics and Biology of Fruit Crop Department, Fondazione Edmund Mach (FEM), Istituto Agrario San Michele (IASMA), Via Mach 1, 38010 San Michele all’Adige, TN Italy; 6 Department of Natural Sciences, University of Maryland Eastern Shore, 1117 Trigg Hall, Princess Anne, 21853 MD USA

**Keywords:** *Mentha aquatica*, *Chrysolina herbacea*, Terpenoids, Insect pheromones, Gut microbial community, Microbial organic volatile compounds, Antimicrobial compounds

## Abstract

**Background:**

A chemical cross-talk between plants and insects is required in order to achieve a successful co-adaptation. In response to herbivory, plants produce specific compounds, and feeding insects respond adequately7 to molecules produced by plants. Here we show the role of the gut microbial community of the mint beetle *Chrysolina herbacea* in the chemical cross-talk with *Mentha aquatica* (or watermint).

**Results:**

By using two-dimensional gas chromatography–mass spectrometry we first evaluated the chemical patterns of both *M. aquatica* leaf and frass volatiles extracted by *C. herbacea* males and females feeding on plants, and observed marked differences between males and females volatiles. The sex-specific chemical pattern of the frass paralleled with sex-specific distribution of cultivable gut bacteria. Indeed, all isolated gut bacteria from females belonged to either α- or γ-Proteobacteria, whilst those from males were γ-Proteobacteria or Firmicutes. We then demonstrated that five *Serratia marcescens* strains from females possessed antibacterial activity against bacteria from males belonging to Firmicutes suggesting competition by production of antimicrobial compounds. By in vitro experiments, we lastly showed that the microbial communities from the two sexes were associated to specific metabolic patterns with respect to their ability to biotransform *M. aquatica* terpenoids, and metabolize them into an array of compounds with possible pheromone activity.

**Conclusions:**

Our data suggest that cultivable gut bacteria of *Chrysolina herbacea* males and females influence the volatile blend of herbivory induced *Mentha aquatica* volatiles in a sex-specific way.

**Electronic supplementary material:**

The online version of this article (doi:10.1186/s12870-017-0986-6) contains supplementary material, which is available to authorized users.

## Background

During the long course of evolution, plants have evolved a wide range of defense mechanisms against herbivores that can be divided into two main categories: pre-formed constitutive defenses and inducible defenses [1]. Physical and chemical barriers existing before insect attack are used as constitutive defenses, whereas direct and indirect defenses are induced defenses that are triggered by the insect attack. Direct defenses are plant traits able to directly interfere with attacking insects, whereas indirect defenses comprise plant traits that do not affect the susceptibility of host plants by themselves, but can serve as attractants to natural enemies of attacking insects [2–7]. The response of insects to plant defenses includes the selective choice of different feeding sites, the alteration of the feeding rate or the induction of physiological/detoxification enzymes [8].

Plants that accumulate specialized metabolites like the aromatic plant *Mentha aquatica* deter herbivory with a direct defense mechanism, by producing constitutively terpenoids in glandular trichomes, which are specialized secretory tissues [5]. Despite the toxic content of such secretory structures, specialized herbivores not only feed on plants bearing these structures, but also have evolved the ability to recognize and being attracted by specific compounds. This kind of feeding adaptation has been described for several insect species [9]. Therefore, interactions between these insects and their host plants occasionally can lead to highly specific relationships, as in the case of *M. aquatica* and *Chrysolina herbacea*.

As demonstrated by Zebelo et al. [10], emission of volatile compounds from the glandular trichomes of *M. aquatica* is mainly characterized by the presence of the monoterpene pulegone. The pulegone, which is the major compound occurring in healthy undamaged leaves, revealed to be a potent attractant to *C. herbacea,* as shown by olfactometry bioassays. The plant response to *C. herbacea* herbivory was the activation of genes for terpenoid biosynthesis, eventually diverting most of monoterpene production from pulegone to the synthesis of menthofuran. The latter compound was found to significantly repel *C. herbacea* in bioassay tests. Despite the presence of lower amounts of many other monoterpenes, no significant difference was found in this group of molecules between infested and uninfested plants, thus confining the deterrent effect mainly to methofuran. As it is typical of plant-insect interactions, mechanical damage was not able to induce in *M. aquatica* the same response as that elicited by *C. herbacea* herbivory. Thus *C. herbacea* is attracted by pulegone produced by undamaged *M. aquatica*, but is deterred by the methofuran production by herbivore-infested *M. aquatica*. These results indicate a differential tolerance of *C. herbacea* to *M. aquatica* monoterpenes; however, the detoxifying mechanisms and the catabolic/biotransforming ability that give the insects the way to tolerate a high amount of ingested terpenoids remain an open question.

Cordero and colleagues [11], by using a combination of headspace solid-phase microextraction (HS-SPME) coupled to comprehensive two-dimensional (2D) gas chromatography combined with mass spectrometry (GCxGC-MS), analyzed the catabolic fate of monoterpenes present in some *Mentha* species by evaluating the terpene content of *C. herbacea* frass (faeces) after feeding on fresh leaves. The carvone-rich *M. spicata* L. [12], the menthol and menthone containing *M. x piperita* L. [13], and a chemotype of *M. longifolia* L., particularly rich in piperitenone oxide [14] were used to demonstrate the ability of *C. herbacea* to metabolize the plant terpenoids, and the insect’s amazing ability to catabolize/biotransform them thereby producing new compounds. For instance, carvone and *Z*-carveol were detected in *C. herbacea* frass after feeding on *M. x piperita*, a mint species that does not accumulate these two terpenes but accumulates limonene [15]. Microorganisms living in the insect intestinal tract might be involved in biotransformation of leaf volatile terpenoids [16]. Indeed, there is clear evidence of microbial transformation of limonene to *Z*-carveol and carvone [17–21]. It is known that microorganisms are able to activate catabolic and metabolic processes that are absent in insects, hence acting as “microbial brokers”, a strategy that enables phytophagous insects hosting such bacteria to overcome biochemical barriers to herbivory [22, 23]. Recently described examples are the detoxification of caffeine by gut microbes of the coffee berry borer [24], the ability of bark beetles bacterial symbionts to metabolize toxic monoterpenes and diterpene acids produced by the mountain pine beetle in response to herbivore damage [25], and the capability of the gut microbiome of cabbage root fly larvae to catalyze the conversion of the plant toxin 2-phenylethyl isothiocianate [26]. Thus, microbial degradation of plant toxic compounds can occur in insect guts and contribute to the carbon and energy requirements for the host [27].

The metabolic activity of insects feeding on plants or/and their associated microorganisms often leads to release of oxygenated derivatives of leaf volatiles. For instance, some insects feeding on plants that, like *M. aquatica*, store 1,8-cineole, a monoterpene oxygenated compound that shows mosquito feeding deterrent and ovipositional repellent activities, and toxic effect against stored-grain beetles [28, 29], show a marked ability to metabolize 1,8-cineole into several hydroxyl derivatives such as 3-hydroxycineol [30, 31]. As Southwell and coworkers [31] suggested, an interesting challenge would be to assess whether 1,8-cineole hydroxylation may represent either a detoxification or a metabolic strategy to produce semiochemicals. Indeed, it has been suggested that several insects, such as possums [32], *Paropsisterna tigrina* [33] and Leichhardt’s grasshopper [34], might use hydroxycineoles as pheromonal markers. This hypothesis could extend the possible role of the microorganisms living in the insect intestinal tract, which could be involved in biosynthesis of semiochemicals.

In this study we have tested this hypothesis by characterizing the cultivable bacterial communities inhabiting the intestinal tract of *C. herbacea* female and male individuals feeding on *M. aquatica*, and then investigating the possibility that the microbial communities from the two sexes could be associated to specific metabolic patterns with respect to the ability to biotransform *M. aquatica* essential oil and release potential semiochemicals.

## Results

### Chemical fingerprint of *M. aquatica* leaf and *C. herbacea* frass volatiles

The discrimination of chemical patterns produced during the interaction between organisms represents one of the major challenges posed by multitrophic interactions [16, 35–38]. The chemical patterns and their contribution to metabolic interactions between *M. aquatica,* its specific herbivore *C. herbacea* and the insect microbial community were here analyzed to elucidate these multitrophic interactions. A clear chemical fingerprint of leaf and insect frass volatiles was found as reported in Table 1. GCxGC-MS analyses allowed the characterization of more than 60 compounds (including green leaf volatiles, mono- and sesquiterpenoids).

**Table 1:** Quantitative descriptors of 2D peaks abundance is reported as 2D normalized volumes % and referred to the TIC current signal; data is the mean of six biological replicates. As expected, the major leaf compound is menthofuran followed by high percentages of its precursor pulegone

Compound name	1D Rt (min)	2D Rt (s)	*I* ^T^s	*I* ^T^sTab	*M. aquatica* leaf	Male frass	Female frass
(*E*)-2-Hexenal	11.69	1.26	859	854	0.01	nd	nd
(*E*)-Hex-3-ene-1-ol	11.75	1.64	860	851	0.04	0.06	0.05
α-Thujene	15.42	0.72	927	931	0.27	0.18	0.20
α-Pinene	15.75	0.76	933	939	1.01	0.38	0.31
Camphene	16.62	0.8	949	953	0.12	0.01	0.02
β-Pinene	18.02	0.88	974	979	1.35	0.33	0.58
1-Octene-3-ol	18.22	1.6	978	978	0.05	0.39	0.15
Sabinene	18.22	0.88	978	975	0.41	0.87	0.97
Myrcene	18.89	0.93	990	991	0.64	0.30	0.39
3-Octanol	19.15	1.52	995	993	0.05	0.17	0.09
α-Phellandrene	19.49	0.84	1001	1005	0.04	nd	nd
(*E*)-Hex-3-enyl-acetate	19.82	1.3	1007	1007	0.02	nd	nd
α-Terpinene	20.42	0.97	1017	1018	0.1	0.02	0.02
*p*-Cymene	20.95	1.14	1027	1026	0.11	0.13	0.12
Limonene	21.22	1.14	1031	1029	1.21	0.86	1.04
1,8-Cineole	21.29	1.09	1033	1031	0.03	6.12	9.64
(*Z*)-β-ocimene	22.22	1.01	1049	1040	0.06	nd	nd
γ-Terpinene	22.75	1.05	1058	1062	0.17	0.05	0.05
*Cis*-Sabinene hydrate	23.42	1.6	1070	1068	0.12	0.00	0.01
α-Terpinolene	24.69	1.01	1092	1088	0.09	0.06	0.02
*p*-Cymenene	24.75	1.3	1093	1089	0.01	nd	nd
2-Nonanol	25.02	1.56	1098	1098	0.03	nd	nd
Linalool	25.29	1.68	1102	1097	1.33	0.37	0.74
Nonanal	25.55	1.52	1107	1098	0.06	0.10	0.04
Allo-ocimene	27.02	1.18	1133	1129	0.03	nd	nd
Unknown#1 (MW152)	27.55	1.3	1142	nn	Nd	0.96	1.26
Unknown#2 (MW152)	27.69	2.06	1144	1140	Nd	1.87	1.01
Menthofuran	29.09	1.39	1169	1164	47.82	23.04	25.71
Isomenthone	29.15	1.77	1170	1164	2.32	1.93	5.74
Unknown#3 (MW152)	29.69	1.6	1179	nn	0.0001	0.18	0.17
α-Terpineol	30.69	1.85	1197	1189	3.71	1.11	2.08
decanal	31.42	1.56	1210	1204	0.06	0.04	0.02
4,7-dimethyl-benzofuran	31.89	1.85	1218	nn	0.13	0.08	0.12
2-α-Hydroxy-1,8-cineole	32.42	2.4	1228	1228	Nd	4.88	3.81
Unknown#4 (MW152)	32.49	2.48	1229	nn	0.14	nd	nd
δ-terpineol	33.22	1.35	1243	1217	0.01	0.31	0.69
3-α-Hydroxy-1,8-cineol	33.35	2.53	1245	1246	0.0001	17.27	11.07
Pulegone	33.49	1.85	1248	1237	17.52	2.47	5.36
3-β-Hydroxy-1,8-cineol	33.55	2.44	1249	1259	Nd	1.57	1.23
Unknown#5 (MW170)	33.89	2.15	1255	1232	Nd	0.60	0.23
Piperitone	34.09	2.36	1259	1252	0.02	nd	nd
9-OH cineole	34.55	2.23	1267	1267	Nd	3.57	1.69
Unknown#8 (MW164)	39.35	2.48	1358	nn	Nd	0.23	0.21
α-Cubebene	39.42	1.26	1359	1351	0.03	nd	nd
α -Copaene	40.55	1.26	1381	1376	0.02	nd	nd
β-Bourbonene	41.35	1.35	1396	1384	0.01	nd	nd
β-Elemene	41.62	1.39	1401	1391	0.05	nd	nd
*Trans*-Jasmone	41.89	2.31	1407	1388	0.07	nd	nd
Unknown#9 (MW166)	41.95	3.03	1408	nn	0.0001	1.04	0.91
α-Gurjunene	42.62	1.3	1422	1409	0.17	nd	nd
β-Cariophyllene	43.15	1.52	1432	1419	0.67	0.24	0.22
Trans-aromadendrene	43.89	1.39	1447	1439	0.07	nd	nd
α-Humulene	44.82	1.47	1466	1454	0.05	nd	nd
Alloaromadendrene	45.29	1.47	1476	1461	0.06	nd	nd
α-Amorphene	45.89	1.39	1488	1485	0.08	nd	nd
Germacrene D	46.22	1.6	1495	1485	0.09	nd	nd
α-Muurolene	46.75	1.47	1506	1499	0.06	nd	nd
Bicyclogermacrene	46.89	1.52	1509	1494	0.25	nd	nd
γ-Cadinene	47.69	1.43	1526	1513	0.34	0.28	0.28
δ-Cadinene	48.09	1.43	1534	1524	0.25	0.13	0.17
Cadina-1,4-diene	48.55	1.47	1544	1534	0.02	nd	nd
α-cadinene	48.82	1.47	1550	1538	0.07	0.02	0.01
Germacrene D-4-ol	50.35	2.1	1583	1576	0.03	nd	nd
β-Eudesmol	51.29	2.1	1620	1649	0.02	nd	nd
α-cadinol	53.55	1.98	1651	1653	0.02	nd	nd

**Fig. 1:**
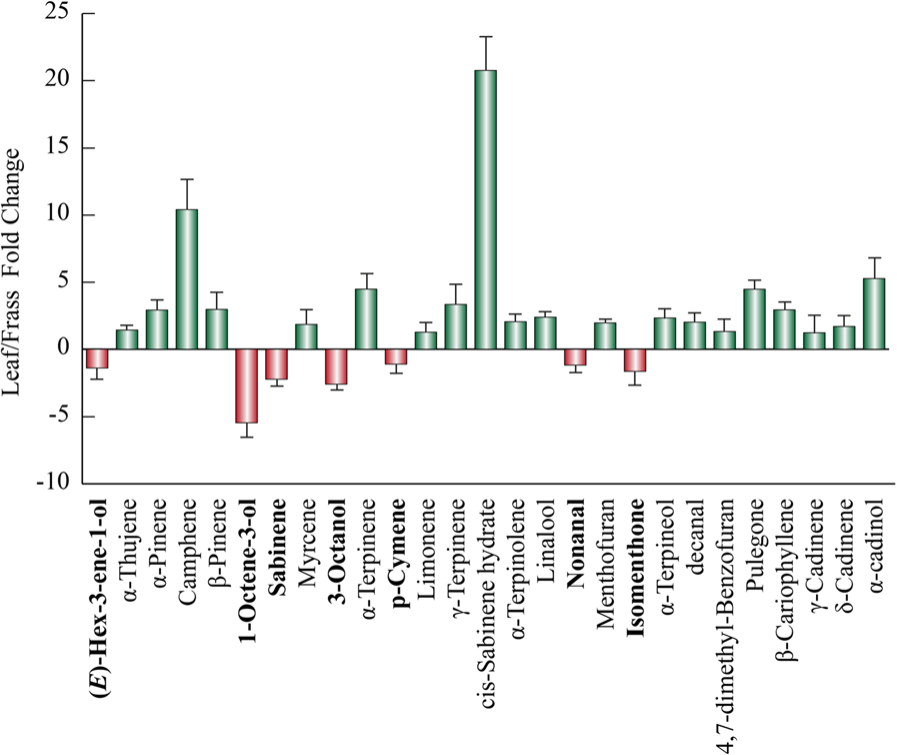
Fold change values discriminating leaf and frass common components in the interaction betweem *Mentha aquatica* and *Chrysolina herbacea*. Orange bars and boldfaced compounds refer to frass extracts. Fold changes are expressed as Leaf value /Frass value. In order to express negative values, data are expressed as −1/(A/B) when the A/B value is <1, where A and B indicate, respectively, Leaf value and Frass value. Error bars represent standard deviations

**Fig. 2:**
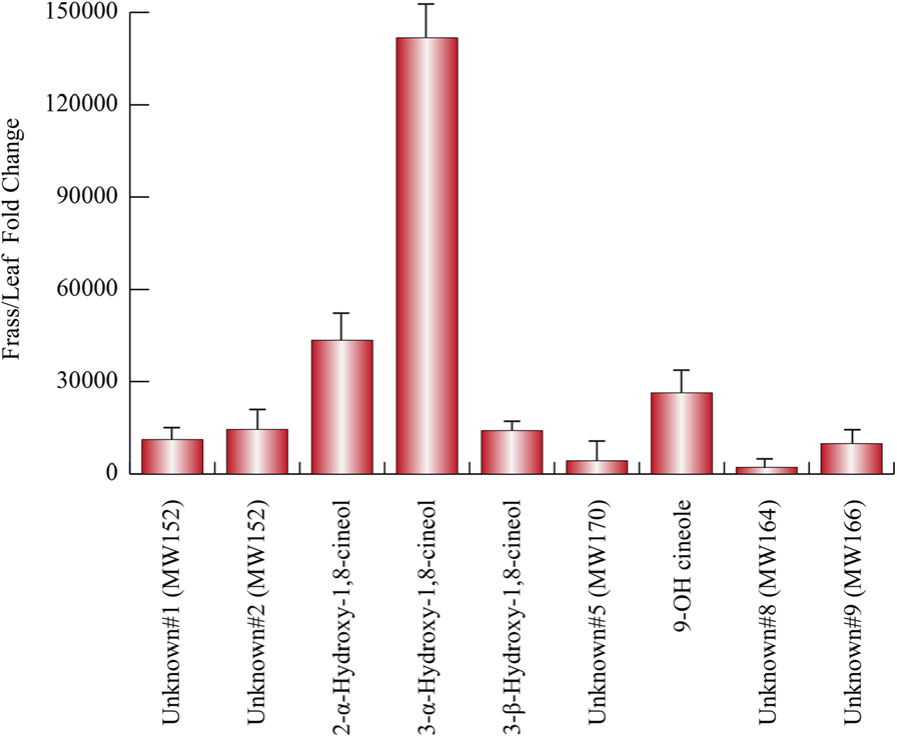
Fold change values discriminating frass and leaf components in the interaction between *Mentha aquatica* and *Chrysolina herbacea*. Fold changes are expressed as Frass value /Leaf value. Error bars represent standard deviations

**Fig. 3:**
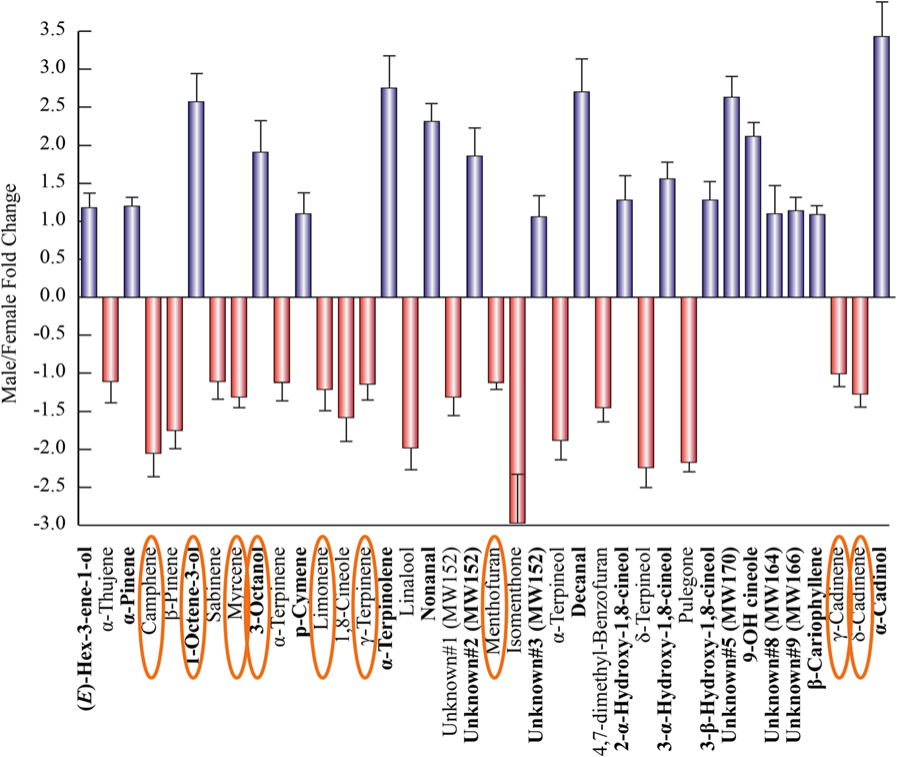
Fold change values discriminating male and female frass components in the interaction betweem *Mentha aquatica* and *Chrysolina herbacea*. Blue bars and boldfaced compounds refer to male extracts. Fold changes are expressed as Male value /Female value. In order to express negative values, data are expressed as −1/(A/B) when the A/B value is <1, where A and B indicate, respectively, Male value and Female value. Error bars represent standard deviations. Compounds that are also indicated in fig. 7 are circled in orange

By using the dataset of Table 1, we calculated the fold change values discriminating leaf and frass components. Considering the leaf components, *cis*-sabinene hydrate, camphene, α-cadinene, α-terpinene and pulegone were more abundant in the leaf, whereas 1-octen-3-ol was particularly present in the frass volatiles (Fig. 1). On the other hand, by considering the characteristic frass compounds we found that four hydroxylated cineole derivatives and five unknown monoterpenes were almost absent in the leaf components (Fig. 2). Differences between male and female volatiles were observed in their frass extracts, with specific chemical patterns including all classes of identified volatile compounds (Fig. 3).

The highly-detailed separation patterns from frass and leaf volatiles were used as chemical signatures for a preliminary fingerprinting investigation aimed at locating informative analytes (fingerprint *minutiae*) whose highly variable abundance between samples (frass vs. leaf) could be considered informative of the interaction between *C. herbacea* and *M. aquatica*. For the preliminary processing of two-dimensional chromatograms a ‘visual’ features approach was used; it consists of a comparative visualization derived from an arithmetic subtraction of a sample (or analyzed) 2D-chromatogram from a reference to reveal compositional differences in the chemical pattern.

**Fig. 4:**
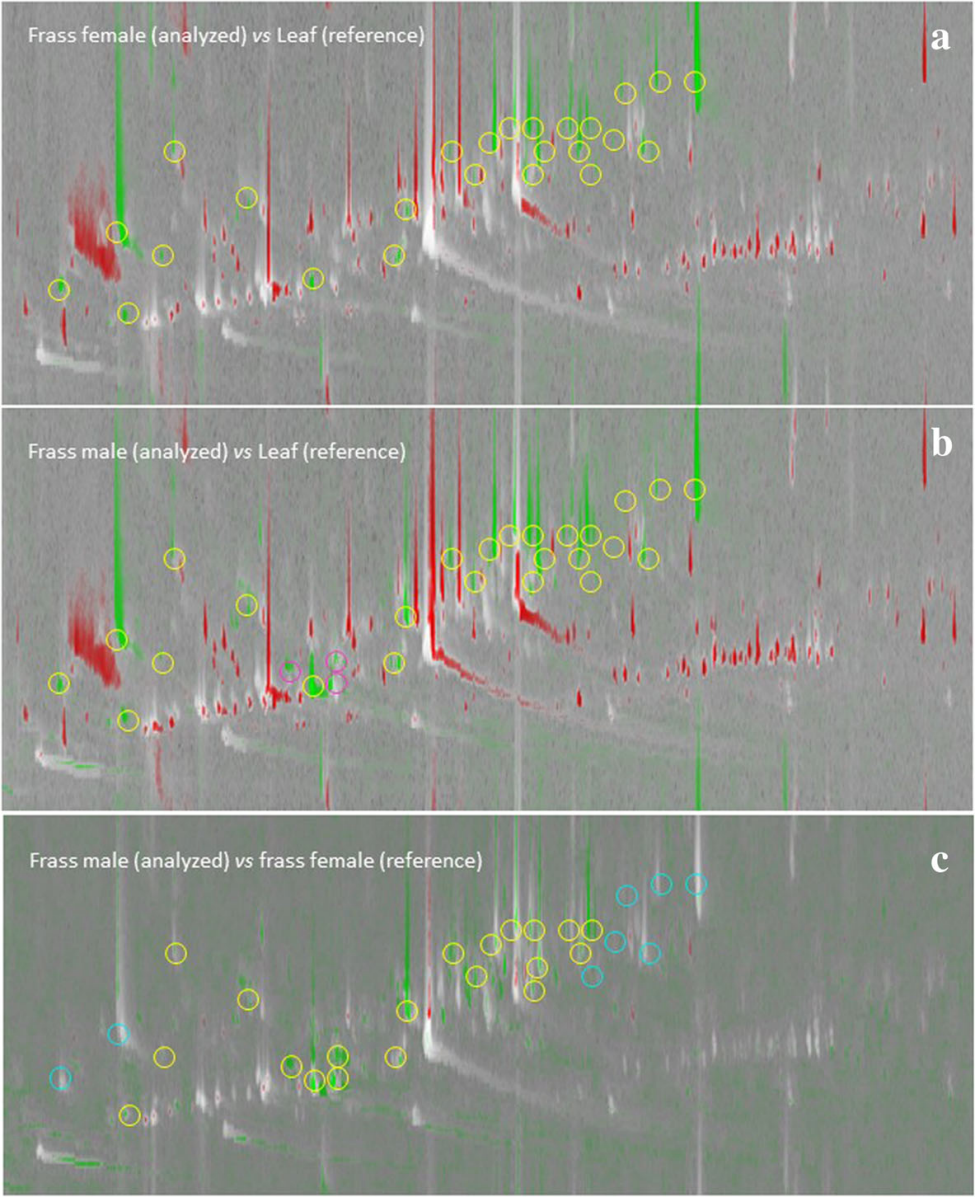
GCxGC two-dimensional analysis pseudocolor comparison of *Mentha aquatica* leaf volatiles and *Chrysolina herbacea* frass volatiles. **a**, pseudocolor comparison of *M. aquatica* leaf volatiles (reference image) compared to the frass volatiles distribution from (**c**). *herbacea* female population feeding on *M. aquatica* leaves. **b**, pseudocolor comparison of *M. aquatica* leaf volatiles (reference image) compared to the frass volatiles distribution from *C. herbacea* male population feeding on *M. aquatica* leaves. **c**, comparative image between females *C. herbacea* frass volatiles (reference image) vs. male *C. herbacea* frass volatiles. See text for explanation

Fig. 4a shows the pseudocolor comparison of *M. aquatica* leaf volatiles (reference image) compared to the frass volatiles distribution from *C. herbacea* female population feeding on *M. aquatica* leaves, whereas Fig. 4b depicts the comparative visualization for *C. herbacea* male population volatiles. Red colorization indicates 2D peaks more abundant in the reference image (i.e., leaf for Figs. 4a and b) while intense green peaks refer to those more abundant in the frass analyzed chromatogram. Yellow circles in Fig. 4a and b indicate *minutiae* features to be investigated as potential bio-transformation and/or degradation products while pink circles indicate analytes gender-specific because of their exclusive presence above the detection limit in male population. Fig. 4c shows the comparative image between frass volatiles from females (reference image) vs. those from males. In this case, the comparative visualization evidences gender-related (semi) quantitative differences between frass volatiles distribution. Light blue circles indicate 2D peaks invariant as a function of *C. herbacea* sex while yellow circles indicate those peaks more abundant in the reference image (i.e., female frass).

### Characterization of the cultivable bacterial communities of the intestinal tract of *C. herbacea* female and male individuals feeding on *M. aquatica*

Gut microorganisms from 10 male and 10 female *C. herbacea* were isolated in pure culture by means of the standard dilution plating technique on Luria-Bertani (LB), Yeast Extract Peptone Dextrose (YEPD) and Nutrient Agar (NA) solid media. Bacterial colonies were visible after cultivation at 30 °C for 24 h. Microbial titers from female intestines were about 10^7^ colony-forming units (CFU)/mL (= per gut) on NA and LB plates, and 10^5^ CFU/mL on YEPD plates. Those from males were 10^7^ CFU/mL on LB and NA plates, while CFU on YEPD plates were about 10^3^ CFU/mL. A total of 245 colonies with distinct morphology, 200 from females and 45 from males (the difference was due to fact that the colonies from females exhibited higher morphological diversity than those from males), were then isolated and examined.

All microbial isolates were preliminary grouped by using the BOX-PCR fingerprinting technique, based on the use of a single BOX-A1R primer which targets the repetitive BOX regions scattered in the genome of bacteria and results in strain-specific fingerprinting [39, 40]. This technique has been successfully used to analyze the microdiversity of bacterial communities [41]. The fingerprints were composed of 8–13 major bands with sizes ranging from about 250 to 3000 bp. Repeatability of the BOX-PCR was as good as the isolates demonstrated identical profiles in three independent experiments. The results of these analyses demonstrated the presence of 10 different genomic patterns among female isolates (Additional file 1: Figure S1a, lanes 1–10) and 6 among male isolates (Additional file 1: Figure S1b, lanes 11–16).

The bacterial isolates were then identified by 16S rRNA-encoding gene sequencing. The gut isolates were designed with the CHF or CHM abbreviations (for *C. herbacea* females or males, source of isolation) followed by a letter and a serial number. Molecular data highlighted that all bacterial isolates from the gut of females were α- or γ-Proteobacteria belonging to the following genera: *Serratia* (CHF-B4, CHF-B16, CHF-B17, CHF-B26, CHF-B37, CHF-G5), *Pantoea* (CHF-G14), *Pseudomonas* (CHF-PG1, CHF-PG3) and *Sphingomonas* (CHF-PG4). In contrast, those from males gut were γ-Proteobacteria of the genera *Pseudomonas* (CHM-N25, CHM-N31) and *Serratia* (CHM-N28) or Firmicutes belonging to the genus *Bacillus* (CHM-L11, CHM-L21, CHM-L22) (Table 2).

**Table 2:** Taxonomic identification of the gut bacterial isolates from females (CHF) and males (CHM) of *C. herbacea* on the basis of the 16S rRNA gene sequencing

Strain designation	Closest relative strain according to Ez-Taxon	Phylum	Accession number of the closest relative strain	16S rRNA similarity (%)
CHF-B4^a^	*Serratia marcescens* subsp. *sakuensis* KRED^T^	γ-Proteobacteria	AB061685	99.12
CHF-B16^a^	*Serratia marcescens* subsp. *marcescens* DSM 30121^T^	γ-Proteobacteria	AJ233431	99.65
CHF-B17^a^	*Serratia marcescens* subsp. *sakuensis* KRED^T^	γ-Proteobacteria	AB061685	99.80
CHF-B26^a^	*Serratia marcescens* subsp. *sakuensis* KRED^T^	γ-Proteobacteria	AB061685	99.89
CHF-B37^a^	*Serratia marcescens* subsp. *sakuensis* KRED^T^	γ-Proteobacteria	AB061685	99.63
CHF-G5^a^	*Serratia marcescens* subsp. *sakuensis* KRED^T^	γ-Proteobacteria	AB061685	99.51
CHF-G14^a^	*Pantoea vagans* LMG 24199^T^	γ-Proteobacteria	EF688012	99.93
CHF-PG1^a^	*Pseudomonas psychrotolerans* C36^T^	γ-Proteobacteria	AJ575816	99.63
CHF-PG3^a^	*Pseudomonas psychrotolerans* C36^T^	γ-Proteobacteria	AJ575816	99.79
CHF-PG4^a^	*Sphingomonas pseudosanguinis* G1-2^T^	α-Proteobacteria	AM412238	97.62
CHM-L11^b^	*Bacillus anthracis* ATCC 14758^T^	Firmicutes	AB190217	99.85
CHM-L21^b^	*Bacillus firmus* NCIMB 9366^T^	Firmicutes	X60616	99.83
CHM-L22^b^	*Bacillus firmus* NCIMB 9366^T^	Firmicutes	X60616	100.00
CHM-N25^b^	*Pseudomonas salomonii* CFBP 2022^T^	γ-Proteobacteria	AY091258	99.49
CHM-N28^b^	*Serratia liquefaciens* ATCC 27592^T^	γ-Proteobacteria	CP006252	99.72
CHM-N31^b^	*Pseudomonas extremorientalis* KMM 3447^T^	γ-Proteobacteria	AF405328	99.20

^a^isolated from females; ^b^isolated from males

**Fig. 5:**
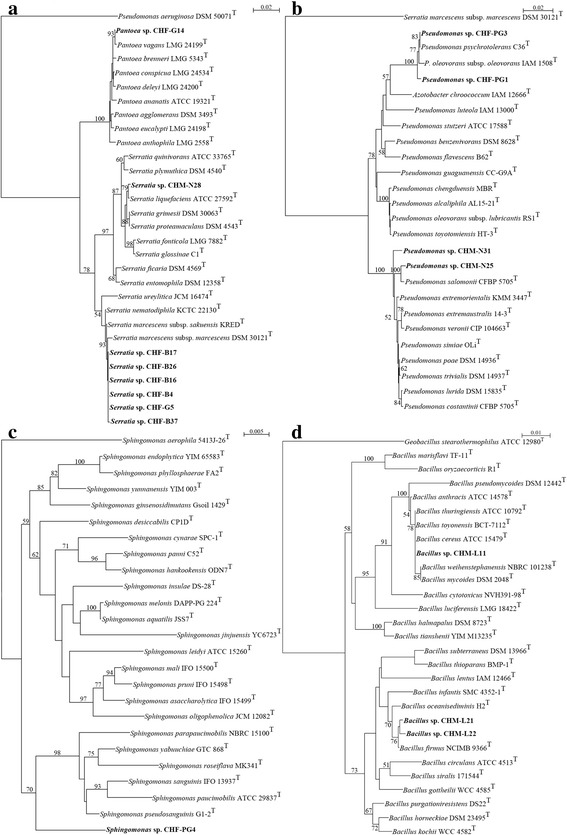
NJ phylogenetic tree based on 16S rRNA gene sequencing of cultivable gut bacteria from females (CHF) and males (CHM) of *C. herbacea*. The phylogenetic relationships of the *Serratia* spp. CHF-B4, CHF-B16, CHF-B17, CHF-B26, CHF-B37, CHF-G5, CHM-N28 and *Pantoea* sp. CHF-G14 (**a**), the *Pseudomonas* spp. CHF-PG1, CHF-PG3, CHM-N25 and CHM-N31 (**b**), the *Sphingomonas* sp. CHF-PG4 (**c**), the *Bacillus* spp. CHM-L11, CHM-L21 and CHM-L22 (**d**) are shown with respect to reference strains. Bootstrap values (expressed as percentages of 1000 replicates) ≥50 are depicted at the branch points. *Pseudomonas aeruginosa* DSM 50071^T^, *Serratia marcescens* subsp. *marcescens* DSM 30121^T^, *Sphingomonas aerophila* 5413 J-26^T^ and *Geobacillus stearothermophilus* ATCC 12980^T^ were used as outgroups in A, B, C and D respectively. Bars, substitutions per nucleotide position

Phylogenetic relationships between the 16S rRNA gene sequences of the cultivable *C. herbacea* gut bacterial communities and those of their closely related reference strains are shown in Fig. 5 a-d. The phylogenetic analysis of the *Enterobacteriaceae* tree (Fig. 5a) demonstrated high similarity between CHF-B4, CHF-B16, CHF-B17, CHF-B26, CHF-37 and CHF-G5 16S rRNA gene sequences and those of *Serratia marcescens* subsp. *sakuensis* KRED^T^ [42] and *S. marcescens* subsp. *marcescens* DSM 30121^T^ [43]. CHM-N28 isolate clustered with the type strain *Serratia liquefaciens* CIP 103238^T^ [44] (Fig. 5a), while the 16S rRNA gene sequence of the isolate CHF-G14 sequence closely allied to that of *Pantoea vagans* LMG 24199^T^ [45] (Fig. 5a).

The phylogenetic analysis of the *Pseudomonadaceae* tree (Fig. 5b) showed that isolates CHF-PG1 and CHF-PG3 branched in a cluster including the reference strains *Pseudomonas psychrotolerans* DSM 15758^T^ [46] and *Pseudomonas oleovorans* subsp. *oleovorans* ATCC 8062^T^ [47]. Furthermore, it collocated the isolates CHM-N25 and CHM-N31 in the *Pseudomonas salomonii* CFBP 2022^T^ cluster [48].

The phylogenetic analysis of the *Sphingomonadaceae* tree (Fig. 5c) highlighted that the isolate CHF-PG4 branched in a new taxonomic cluster. Indeed, its 16S rRNA gene sequence showed a low similarity (97.62%) with that of the reference strain *Sphingomonas pseudosanguinis* G1-2^T^ [49] (Table 2).

Lastly, phylogenetic data collocated CHM-L11 in the *Bacillus cereus* group including eight closely related species: *B. cereus* [50], *B. anthracis* [51], *B. thuringiensis* [52], *B. toyonensis* [53], *B. mycoides* [54], *B. pseudomycoides* [55], *B. weihenstephanensis* [56] and *B. cytotoxicus* [57] (Fig. 5d). CHM-L21 and CHM-L22 clustered together with the reference strain *Bacillus firmus* NCIMB 9366^T^ [58] (Fig. 5d).

### Limited culture-independent molecular analysis of gut-associated bacterial communities

**Fig. 6:**
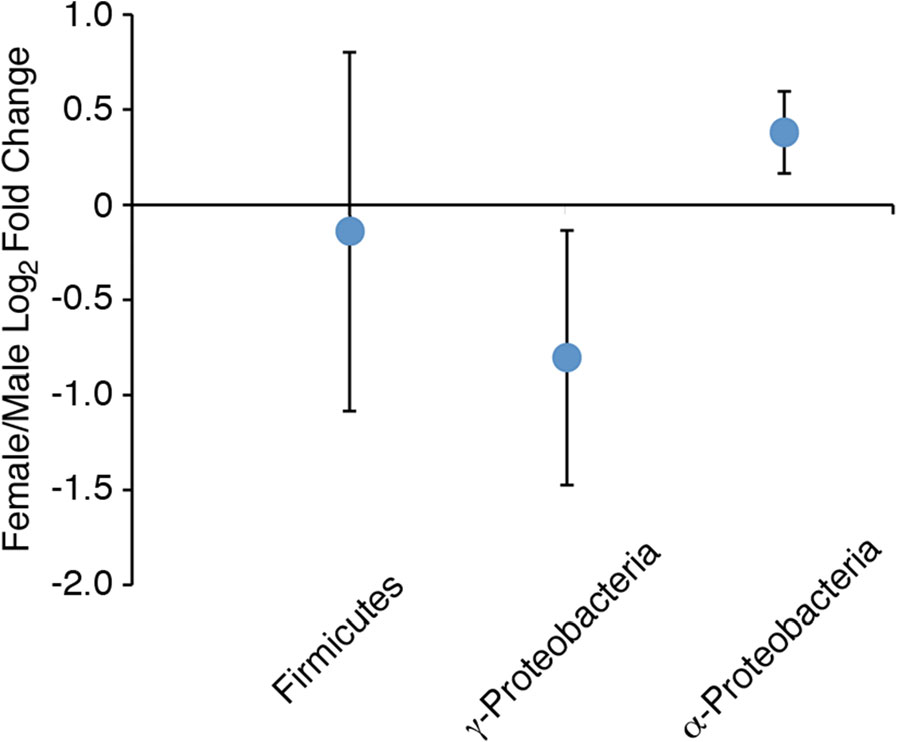
Limited culture-independent analysis of *C. herbacea* male and female gut bacteria. Relative abundance of Firmicutes, α- and γ-Proteobacteria 16S rRNA gene sequences in *C. herbacea* male and female guts was determined by real-time PCR using phylum/subphylum specific primer pairs. Results are expressed as female/male log_2_ fold changes (circles) ± standard deviations (error bars)

The sex-specific distribution of cultivable gut bacteria led us to explore the bacterial community diversity by limited culture-independent molecular analysis targeting 16S rRNA gene sequences from Firmicutes, α- and γ-Proteobacteria. The analysis was carried out by firstly amplifying 16S rRNA gene sequences from male and female gut communities using bacterial broad-range primer pairs in standard PCR, and then analyzing the relative amounts of amplifiable Firmicutes, α- and γ-Proteobacteria 16S rRNA gene sequences by semi-quantitative real-time PCR using phylum/subphylum-specific primer pairs (Fig. 6 and Additional file 2: File S1). In agreement with culture-dependent analysis, the results of real-time PCR experiments confirmed the presence of γ-Proteobacteria 16S rRNA gene sequences in all samples with quantitative values higher in samples from male than from female individuals (Fig. 6 and Additional file 2: File S1). Moreover, real time PCR amplification with primers specific for α-Proteobacteria, which could not be isolated from males, provided quantitative values lower in samples from males than from females individuals. However, in contrast with the data of culture-dependent analysis showing the absence of Firmicutes in the cultivated gut community from females, real time PCR amplification with Firmicutes-specific primers produced amplifiable signals in the DNA samples from both males and females with almost similar quantitative values in both sexes. This discrepancy can be traced to possible sex-specific differences in the structures of Firmicutes from males and females, and to unsuitable cultivation conditions for isolation of several members of Firmicutes (including anaerobic strains) whose presence could be detected by real-time PCR.

### Cross-inhibition tests with isolated gut bacteria from *C. herbacea* female and male individuals

The qualitative difference in the cultivable gut bacterial community found in the two sexes may reflect the sexual dimorphism (females are typically larger than males) of *C. herbacea* due to anatomic differences in the gut. However, to better understand this aspect we tested a possible growth incompatibility among the isolates by performing a series of cross-inhibition tests as detailed in Methods (Table 3 and Additional file 1: Figure S2). As shown in Table 3, five gut isolates from *C. herbacea* females (CHF-B4, CHF-B16, CHF-B17, CHF-B26 and CHF-B37), phylogenetically related to the γ-Proteobacterium *Serratia marcescens* subsp. *sakuensis*, determined zones of growth inhibition on three isolates from males (CHM-L11, CHM-L21, CHM-L22) belonging to the genus *Bacillus* of the Firmicutes group with diameters ranging from 0.52 to 3.52 cm. In addition, these five bacteria possessed an antibacterial activity against three others Gram-positive tester microorganisms such as *Staphylococcus aureus* [59], *Micrococcus luteus* [60] and the actinomycete *Streptomyces ambofaciens* [61]. The assayed microorganisms were able to produce zones of growth inhibition on tester strains with diameters between 0.90 and 2.93 cm (Table 3). In contrast, they did not show any antibacterial effect on either γ-Proteobacterial isolates from males (CHM-N25, CHM-N28 and CHM-N31) or two γ-Proteobacterial tester microorganisms, *E. coli* [62] and *S. enterica* [63]. Conversely, no gut strain isolated from males showed detectable antibacterial activity against isolates from females and tester microorganisms. Notably, the rifamycin B producer *A. mediterranei* S699 showed diameters of growth inhibition against the three Gram-positive isolates from males (CHM-L11, CHM-L21, CHM-L22) ranging from 2.2 and 2.8 cm. These inhibition values were similar to those found with the five gut isolates from females (CHF-B4, CHF-B16, CHF-B17, CHF-B26 and CHF-B37) supporting their strong antibacterial activity.

**Table 3:** Cross-inhibition tests showing antibacterial activities of bacterial isolates from females against bacterial isolates from males and reference strains^a^

Assayed strain^b^	Tester strain^c^										
	*Bacillus* sp. CHM-L11	*Bacillus* sp. CHM-L21	*Bacillus* sp. CHM-L22	*Pseudomonas* sp. CHM-N25	*Serratia* sp. CHM-N28	*Pseudomonas* sp. CHM-N31	*Escherichia* *coli* FB8	*Salmonella typhimurium* LT2	*Staphylococcus* *aureus* SA-1	*Micrococcus* *luteus* ML-1	*Streptomyces* *ambofaciens* ATCC 23877
*Serratia* sp. CHF-B4	1.21 ± 0.18	2.03 ± 0.11	2.04 ± 0.09	N.I.	N.I.	N.I.	N.I.	N.I.	1.83 ± 0.30	1.77 ± 0.25	2.93 ± 0.51
*Serratia* sp. CHF-B16	0.54 ± 0.09	1.13 ± 0.10	1.5 ± 0.12	N.I.	N.I.	N.I.	N.I.	N.I.	1.27 ± 0.25	0.90 ± 0.36	2.57 ± 0.42
*Serratia* sp. CHF-B17	1.18 ± 0.22	2.98 ± 0.18	2.80 ± 0.30	N.I.	N.I.	N.I.	N.I.	N.I.	1.03 ± 0.20	1.18 ± 0.28	1.17 ± 0.28
*Serratia* sp. CHF-B26	0.52 ± 0.05	3.15 ± 0.13	3.03 ± 0.15	N.I.	N.I.	N.I.	N.I.	N.I.	2.53 ± 0.50	1.77 ± 0.25	2.67 ± 0.29
*Serratia* sp. CHF-B37	0.55 ± 0.06	3.52 ± 0.10	3.05 ± 0.14	N.I.	N.I.	N.I.	N.I.	N.I.	2.67 ± 0.57	2.33 ± 0.28	1.28 ± 0.20
*Serratia* sp. CHF-G5	N.I.	N.I.	N.I.	N.I.	N.I.	N.I.	N.I.	N.I.	N.I.	N.I.	N.I.
*Pantoea* sp. CHF-G14	N.I.	N.I.	N.I.	N.I.	N.I.	N.I.	N.I.	N.I.	N.I.	N.I.	N.I.
*Pseudomonas* sp. CHF-PG1	N.I.	N.I.	N.I.	N.I.	N.I.	N.I.	N.I.	N.I.	N.I.	N.I.	N.I.
*Pseudomonas* sp. CHF-PG3	N.I.	N.I.	N.I.	N.I.	N.I.	N.I.	N.I.	N.I.	N.I.	N.I.	N.I.
*Sphingomonas* sp. CHF-PG4	N.I.	N.I.	N.I.	N.I.	N.I.	N.I.	N.I.	N.I.	N.I.	N.I.	N.I.

^a^ The diameters (cm) of zones of growth inhibition were measured after subtracting the 1 cm-diameter of the agar disk containing the assayed bacterium, Values are presented as mean ± SD. N.I., no detectable inhibition

^b^Assayed strain, bacterium producing a diffusible compound with a possible antibacterial activity

^c^Tester strain, bacterium used to detect antibiotic activity

### Ability of gut bacteria to metabolize *M. aquatica* essential oil

**Fig. 7:**
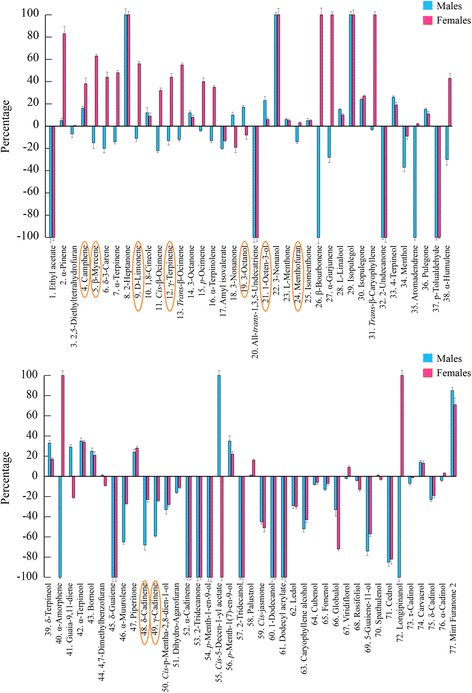
Biotransformation of *M. aquatica* essential oil by *C. herbacea* sex-specific gut microbial communities. *M. aquatica* essential oil was incubated with gut bacteria co-cultures from *C. herbacea* male and female individuals, and male (blue)- and female (red)-specific terpene profiles were determined by SBSE and GC-MS. Values are expressed as percentage increase/decrease with respect to the control without bacteria. Detected compounds: 1, ethyl acetate; 2, α-pinene; 3, 2,5-diethyltetrahydrofuran; 4, camphene; 5, β-myrcene; 6, δ-3-carene; 7, α-terpinene; 8, 2-heptanone; 9, (−)-limonene; 10, 1,8-cineole; 11, cis-β-ocimene; 12, γ-terpinene; 13, trans-β-ocimene; 14, 3-octanone; 15, p-ocimene; 16, α-terpinolene; 17, amyl isovalerate; 18, 3-nonanone; 19, 3-octanol; 20, all-trans-1,3,5-undecatriene; 21, 1-octen-3-ol; 22, 3-nonanol; 23, (−)-menthone; 24, (+)-menthofuran; 25, isomenthone; 26, β-bourbonene; 27, α-gurjunene; 28, L-linalool, 29, isopulegol; 30, isopulegone; 31, trans-β-caryophyllene; 32, 2-undecanone; 33, 4-terpineol; 34, menthol; 35, aromadendrene; 36, (+)-pulegone; 37, p-tolualdehyde; 38, α-humulene; 39, δ-terpineol; 40, α-amorphene; 41, guaia-9,11-diene; 42, α-terpineol; 43, borneol; 44, 4,7-dimethylbenzofuran; 45, δ-guaiene; 46, α-muurolene; 47, piperitone; 48, δ-cadinene; 49, γ-cadinene; 50, cis-p-mentha-2,8-dien-1-ol; 51, dihydro-agarofuran; 52, α-cadinene; 53, 2-tridecanone; 54, p-menth-1-en-9-ol; 55, cis-5-decen-1-yl acetate; 56, p-menth-1(7)-en-9-ol; 57, 2-tridecanol; 58, palustrol; 59, cis-jasmone; 60, 1-dodecanol; 61, dodecyl acrylate; 62, ledol; 63, caryophyllene alcohol; 64, cubenol; 65, fonenol; 66, globulol; 67, viridiflorol; 68, rosifoliol; 69, 5-guaiene-11-ol; 70, spathulenol; 71, cedrol; 72, longipinanol; 73, τ-cadinol; 74, carvacrol; 75, δ-cadinol; 76, α-cadinol; 77, mint furanone 2. Error bars represent standard deviations. Compounds that are also indicated in Fig. 3 are circled in orange

In order to clarify the possible role of the gut bacterial communities from *C. herbacea* female and male individuals in the adaptation of *C. herbacea* to *M. aquatica* volatiles, we assayed the ability of these isolated gut bacteria to grow either individually or in community utilizing essential oil as the sole carbon (and energy) source. All 16 bacterial isolates were able to grow well either on solid or in liquid SRM-oil media. In particular, in liquid SRM-oil media, all tested bacteria reached a mean value of 1.0 OD_600_/mL after an incubation time of 24 h at 30 °C (data not shown). The ability of the gut bacteria to grow individually using the essential oil as sole carbon source prompted us to investigate the possible modification of the essential oil components carried out by the two bacterial communities using an in vitro approach. Bacteria from males or females were co-cultivated in the presence of *M. aquatica* essential oil as detailed in the Methods section, and then essential oil components were extracted from the exhausted growth medium by Stir Bar Sorptive Extraction (SBSE) technique and subjected to quantitative analysis by Gas chromatography–mass spectrometry (GC-MS) (Fig. 7).

GC-MS analysis highlighted the ability of gut bacteria to modify the terpene composition of the *M. aquatica* essential oil as compared to controls without bacteria (Fig. 7). 3-nonanol (#22), β-bourbonene (#26), isopulegol (#29), α-amorphene (#40) and longipinanol (#72) were exclusively detected in the females gut bacterial community supernatant (Fig. 7). In contrast, 3-nonanol (#22) and isopulegol (#29) were the new compounds revealed in the male community supernatant (Fig. 7). These compounds were totally absent in the control essential oil (Fig. 7). Furthermore, the two communities were able to alter the main essential oil components such as menthofuran (#24), menthone (#23), 1,8-cineole (#10), limonene (#9) and pulegone (#36). As shown in Fig. 7, menthofuran was degraded both by females and males gut bacterial communities. In contrast, with reference to the compounds menthone, 1,8-cineole and pulegone, both gut communities were able to enhance their concentration with respect to the control essential oil. Whilst D-limonene was degraded by male community, its level was enhanced by metabolic activity of the female community. These results were indicative of a potential ability of the gut bacteria to modify the terpene profile of *M. aquatica* essential oil thereby modulating the plant-insect interaction.

## Discussion

The interaction between bacteria and insects is becoming a major topic of discussion, particularly when bacteria contribute to insect fitness through their involvement in food digestion. This interaction provides insect with better nutrition, detoxification, pheromone production, regulation of pH, synthesis of vitamins and sterols, temperature tolerance, resistance against pathogens and parasitoids. Gut bacteria can even modify the use of host plants by phytophagous insects [22, 64–66], thereby affecting insects development, defense against natural enemies, immunity, reproduction, and speciation [22, 67–73].

In the current study, the role of the cultivable gut bacteria of *C. herbacea* male and female individuals in the high specific relationship between this herbivore and *M. aquatica* was investigated. By using a combination of chemical pattern and culture-dependent approach, we found marked qualitative differences in the two sexes. It is interesting to observe, as preliminary information about differential metabolism, that female frass analytes (30.05 μg/g ± 2.63) are present in a higher concentration with respect to male frass analytes (17.41 μg/g ± 2.05). The algorithm enabling the highly reliable and specific fingerprinting approach used in this work has been demonstrated to be successful for complex patterns investigations: including breast cancer metabolomics [74], bio-oils characterization [75] and mice urine metabolite profiling [76], whereas the peak-region features approach consists of a sequence of operations as previously detailed [77–80].

The sex-specific chemical pattern of the frass paralleled with sex-specific distribution of cultivable gut bacteria. The bacterial isolation procedure revealed the selective presence of α- and γ-Proteobacteria in the intestinal tract of females; in contrast, the gut of males contained both γ-Proteobacteria and Firmicutes (Table 2). This result was only partially consistent with that of limited culture-independent molecular analysis targeting 16S rRNA gene sequences from Firmicutes, α- and γ-Proteobacteria (Fig. 6 and Additional file 2: File S1). The analysis confirmed the presence of γ-Proteobacteria in both male and female intestines, and demonstrated lower abundance of α-Proteobacteria in the cultivable gut community from males. However, it revealed a large presence of Firmicutes in male intestine emphasizing the well-known limits of culture-based methods.

Most of the gut bacterial isolates from the two sexes are taxonomically related to species previously found as insect gut bacteria [81]. The isolates CHF-B4, CHF-B16, CHF-B17, CHF-B26, CHF-B37 and CHF-G5 from females are phylogenetically close to *S. marcescens* (Fig. 5a), a γ-Proteobacterium associated to numerous species and genera of the order Orthoptera (crickets and grasshoppers), Isoptera (termites), Coleoptera (beetles and weevils), Lepidoptera (moths), Hymenoptera (bees and wasps), and Diptera (flies) [81]. CHM-N28 isolate from males is phylogenetically identical to *S. liquefaciens* (Fig. 5a), found in association with *S. marcescens* in sugar-beet root-maggot (*Tetanops myopaeformis*) developmental stages, suggesting an insect-microbe symbiosis, as well as a nutritional interdependence [82]. On the other hand, *S. marcescens* and *S. liquefaciens* are also considered potential insect pathogens for more than 70 species [83]. As demonstrated by Stephens [84], the hemolymph of insects, which is normally bactericidal for nonpathogens, cannot prevent multiplication of potential pathogens. Antibacterial substances in ingested leaves might interfere with bacterial multiplication, but strains of this genus were found resistant to these [85]. Moreover, lecithinase, proteinase, and chitinase play a crucial role in the virulence of *Serratia* for insects, and purified *Serratia* proteinase or chitinase is very toxic when injected into the hemocoel [86, 87].

CHF-G14 isolate is phylogenetically related to the Gram-negative γ-Proteobacterium *P. vagans* (Fig. 5a). *P. vagans* strain C9-1 was found recently associated with the fungus-growing ants [88]. CHF-PG1 and CHF-PG3 on the basis of the 16S rRNA gene sequencing appeared to be close to *Pseudomonas psychrotolerans* and *P. oleovorans* subsp. *oleovorans* (Fig. 5B), while, the isolates CHM-N25 and CHM-N31 were strictly related to *P. salomonii* (Fig. 5B). To our knowledge, these species of the genus *Pseudomonas* were never found as insect gut colonizers. Molecular data showed that the isolate CHF-PG4 did not belong to a previously characterized species of the genus *Sphingomonas* and occupied a distinct taxonomical position (Fig. 5C). *Sphingomonas* spp. were isolated from guts of *Manduca sexta* [89], *Anopheles stephensi* [90], *Helicoverpa armigera* [91] and *Homalodisca vitripennis* [92]. Lastly, CHM-L11 was taxonomically near to *B. thuringiensis*; whilst CHM-L21 and CHM-L22 were biochemically and phylogenetically identified as *B. firmus* strains (Fig. 5d). Strains of *B. thuringiensis* have been isolated worldwide from many habitats including soil, insects, stored-product dust, and deciduous and coniferous leaves [93]. Notably, *B. thuringiensis* proliferates in the guts of various insects before killing its hosts [94]. *B. firmus* was isolated, by using cultivable techniques, from guts of the lepidopteran pest *Helicoverpa armigera* [91].

In order to better clarify why the bacterial communities were qualitatively different, we tested their possible growth incompatibility. We demonstrated that the five gut isolates from females (CHF-B4, CHF-B16, CHF-B17, CHF-B26, CHF-B37) that were taxonomically and biochemically identified as non-pigmented *S. marcescens* strains had an antibacterial activity against isolates from males belonging to the genus *Bacillus* (CHM-L11, CHM-L21, CHM-L22) (Additional file 1: Figure S2). A similar antibacterial activity was found against the other Gram-positive bacteria used as tester microorganisms including *S. aureus*, *M. luteus* and *S. ambofaciens* (Table 3). In all other cases, no detectable growth inhibition was found. These results are consistent with the finding that no strains belonging to *S. marcescens* were isolated from male guts, where many Gram-positive bacteria were found.

We provide evidence that the gut bacteria of *C. herbacea* are able to metabolize *M. aquatica* essential oil components. Interestingly, a correlation was found between the pattern of frass volatiles distribution in male and female individuals (Fig. 3) and that of bio-transformed oil compounds by the cultivable microbial communities (Fig. 7). In particular, 9 out of 32 (about 28%) identified frass volatiles (i.e., camphene, 1-octene-3-ol, myrcene, 3-octanol, limonene, γ-terpinene, menthofuran, γ-cadinene and δ-cadinene; circled in orange in Figs. 3 and 7) exhibited differential increase/decrease percentage in biotransformation experiments in agreement with their relative abundance in frass. This result may provide an indirect measure of how the cultivated microbial community is representative of the actual *in situ* community.

The two bacterial communities from both female and male individuals were able to utilize and biotransform in vitro many terpenoids into an array of new compounds, which were absent in control samples (Fig. 7). In particular, five (3-nonanol, β-bourbonene, isopulegol, α-amorphene, longipinanol) and two (3-nonanol, β-bourbonene) new compounds were detected in the exhausted media from females and males bacterial communities, respectively. 3-nonanol (compound #22 in Fig. 7) has been found in the mandibular gland secretions of the ant *Crematogaster sjostedti*, which could facilitate species identification and lead to species-specific alarm and defense responses that influenced their competitive interactions [95]. β-bourbonene (#26 in Fig. 7) was found to be a plant-derived pheromone compound [96] released by the insect herbivore *Euceraphis punctipennis* [97]. Isopulegol (#29 in Fig. 7) is a pheromone [96] tracked in the pygidial gland secretions of the ant *Azteca chartifex* [98]. α-amorphene (#40 in Fig. 7) is present in the larval osmeterial secretions produced by disturbed *Pachliopta aristolochiae* individuals in order to stave off numerous predators [99]. Numerous published studies have demonstrated the role of bacteria in plant terpene and terpenoid metabolism [100–105]. Although we only focused on cultivable gut bacteria, we hypothesize that these new volatile organic compounds, possibly derived from plant terpenoids as a consequence of gut bacterial metabolisms, could be used as recruitment signals (produced by both bacterial communities) or sex pheromones (produced by the bacterial community from female individuals) by insects. In this regard it is noteworthy that the cultivated bacterial community from female individuals produced a more varied blend than that from males including new compounds (i.e., that were absent in control samples) with potential sex pheromone activity (β-bourbonene [#26 in Fig. 7], α-amorphene [#40 in Fig. 7]).

In addition, we found that the two bacterial communities altered the profile of the two main essential oil components menthofuran (#24) and pulegone (#36). Both bacterial communities degraded menthofuran and enhanced the concentration of pulegone with respect to the control essential oil (Fig. 7). Pulegone is a terpene produced by intact *M. aquatica* plants to attract individuals of *C. herbacea*; in contrast, menthofuran is produced by infested plants and has a potent repellent activity on *C. herbacea* [10].

## Conclusions

This study focuses on the multitrophic association among *M. aquatica*, its specific herbivore *C. herbacea*, and the insect gut microbial community. The results support the evidence that, by modulating the plant-derived terpenoid profile, gut bacteria of *C. herbacea* play a crucial role in co-adaptation between plants and insects enabling a highly specific relationship between *M. aquatica* and *C. herbacea*. Furthermore, these microbial volatile organic compounds (MVOCs) could be exploited as an eco-friendly, cost-effective, and sustainable strategy for agricultural practices [16, 35].

## Methods

### Plant and animal material

Stolons of *Mentha aquatica* L. were collected from wild populations growing in Cambiano (Turin province, Italy, alt 240 m a.s.l.). Plants were grown as reported earlier [10]. Adults of *Chrysolina herbacea* (Duftschmidt 1825) (Coleoptera, Chrysomelidae, Chrysomelinae) were collected by hand picking from infested *M. aquatica* fields. After collection, beetles were reared at 22 °C in ventilated glass chambers and fed with *M. aquatica* cuttings. The beetles were starved for 24 h prior the experiments.

### Essential oil distillation

One Kg of fresh leaves collected from *M. aquatica* cuttings were hydrodistilled with a modified Clevenger apparatus [106] and the essential oil was collected, dried over anhydrous ammonium sulphate and stored at 5 °C for further studies.

### Collection of plant volatiles from *M. aquatica* leaves and *C. herbacea* frass

Fresh leaves (about 50 mg) from living plants of *M. aquatica* were carefully picked-off immediately before the analysis and gently placed in hermetically sealed 2.0 mL vials for HS-SPME sampling. Frass fluid collected from *Chrysolina herbacea* populations reared on *M. aquatica* leaves was immediately transferred to exactly weighted 2.0 mL headspace vials and left to ambient conditions for a fixed time. After 30 min, vials were exactly weighted (to estimate the loss of weight due to water/sample evaporation) and hermetically sealed before HS-SPME sampling. Divinylbenzene/Carboxen/Polydimethylsiloxane (DVB/CAR/PDMS) df 50/30 μm, 2 cm length SPME fiber device was manually inserted into the sealed vial and the fiber exposed to the matrix headspace for volatile sampling. The Internal Standard (ISTD) loading procedure onto the SPME device was performed as previously described [77] using α-thujone. Then, the fiber was exposed to the headspace at room temperature for 20 min. After the ISTD loading, the fiber was exposed to the frass headspace again for 20 min at room temperature.

Frass analyte concentration was calculated based on external calibration curves using mono and sesquiterpenes.

### GC × GC-MS analysis of volatiles

GC × GC analyses were carried out on an Agilent 6890 GC unit coupled with an Agilent 5975 MS detector operating in EI mode at 70 eV (Agilent, Little Falls, DE, USA). Transfer line was set at 270 °C, and ion source at 230 °C. A Standard Tune option was used with a scan range of 35–250 m/z at 12,500 amu/s and a resulting acquisition frequency of 30 Hz. The system was provided with a two-stage thermal modulator (KT 2004 loop modulator from Zoex Corporation, Houston, TX, USA) cooled with liquid nitrogen and with the hot jet pulse time set at 250 ms with modulation time of 4 s. The hot-jet temperature programme was from 160 °C to 250 °C at 3 °C/min. A deactivated fused silica loop of 1.0 m × 0.10 mm *d*_*c*_ was used. GC × GC column set consisted of a ^1^D SE52 column (95% polydimethylsiloxane, 5% phenyl) (30 m × 0.25 mm *d*_*c*_, 0.25 μm *d*_*f*_) coupled with a ^2^D OV1701 column (86% polydimethylsiloxane, 7% phenyl, 7% cyanopropyl) (1 m × 0.1 mm *d*_*c*_, 0.10 μm *d*_*f*_); columns were from MEGA (Legnano (Milan)-Italy). GC S/SL injector: 1/10 split mode; temperature: 250 °C; carrier gas: helium at a constant flow and initial head pressure 280 KPa; Oven programming: from 45 °C (1 min) to 260 °C (5 min) at 2.5 °C/min. Data were acquired by Agilent–MSD Chem Station ver D.02.00.275 (Agilent Technologies, Little Falls, DE, USA) and processed using GC Image software, ver 2.5 (GC Image, LLC Lincoln NE, USA).

### GC-MS analysis of essential oils

Essential oils were analyzed by GC-MS with a system consisting of an Agilent 6890 N GC unit coupled with a 5973A MS detector operating in EI mode at 70 eV (Agilent, Little Falls, DE, USA). Transfer line was set at 280 °C. A Standard Tune option was used with a scan range of 35–250 m/z. The GC column was a ZB-5MS Zebron (Phenomenex, Torrance, CA, US) (95% polydimethylsiloxane, 5% phenyl) (30 m × 0.25 mm *d*_*c*_, 0.25 μm *d*_*f*_). GC S/SL injector: in splitless mode; temperature: 250 °C; carrier gas: helium at a constant flow 1.0 mL/min; Oven programming: from 60 °C (51 min) to 270 °C (5 min) at 3 °C/min.

### Microbiological media

LB (10.0 g/L NaCl, 10.0 g/L tryptone, 5.0 g/L yeast extract, 15.0 g/L agar), YEPD (10.0 g/L yeast extract, 20.0 g/L peptone, 20.0 g/L D-glucose, 20.0/g L agar) and NA (5.0 g/L tryptone, 3.0 g/L beef extract, 15.0 g/L NaCl, 15.0 g/L agar) solid media were used for isolation of gut microorganisms from both female and male individuals of *C. herbacea*. The chemically defined SRM medium (1.0 g/L NH_4_H_2_PO_4_, 0.2 g/L KCl, 0.2 g/L MgSO_4_, 1.0 g/L glucose, 15.0 g/L agar, when requested) was used as a base to formulate either SRM-0 medium (without glucose) or SRM-oil medium by replacing glucose with an emulsion *M. aquatica* essential oil:DMSO (0.002%:0.25%, [v:v]) to test the bacterial growth in the presence of essential oil as sole carbon and energy source. LB, NA, Hickey-Tresner (HT) (1.0 g/L yeast extract, 1.0 g/L beef extract, 2.0 g/L N-Z amine A, 10.0 g/L starch, 15.0 g/L agar) and GYM (4.0 g/L glucose, 4.0 g/L yeast extract, 10.0 malt extract, 2.0 g/L CaCO3, agar 15 g/L) solid media were used for microbiological assays.

### Isolation of the cultivable gut bacteria from *C. herbacea* female and male individuals

Two insect pools (males and females), each consisting of 10 individuals, were placed in a sterile physiological solution (0.9% NaCl). Each insect pool suspension was decanted and rinsed thrice with 10 mL sterile 0.9% (w/v) NaCl solution. Then, the rinsed insect pool was treated with 3% (v/v) H_2_O_2_ for 20 s, and finally rinsed with 70% (v/v) ethanol and rapidly flamed. The throat of each insect was cut with a sterile scalpel and the head was removed. Pressing on the paunch of the cut insects the total intestine was collected in 5 mL LB broth containing 2–3 g of sterile glass beads (ϕ 0.5 mm), heavily vortexed for 4–5 min and left to elute overnight at 4 °C. To remove gut debris, samples were centrifuged at 2000 × g for 1 min. Then, 1:10 serial dilutions of the supernatant were transferred on the surface of LB, NA and YEPD solid media and plated. Samples were incubated under aerobic or microaerophilic condition for 24 h at 30 °C. After this incubation time, a number of colonies with distinct morphology were picked up from each agar plate media and streaked onto fresh plates. Only plates with colony numbers ranging from 50 to 200 were used for isolation of pure cultures. Pure cultures were checked by microscopy, and stored either in above mentioned agar slants or in broth plus 20% (v/v) glycerol at −80 °C.

### DNA procedures

All gut bacteria (200 from females and 45 from males) were grown in 20 mL of the above-mentioned liquid media with rotary shaking to late logarithmic phase. After centrifugation at 2000 × g for 20 min, pellets were re-suspended in 500 μL of SET buffer (75 mM NaCl, 25 mM EDTA, 20 mM Tris–HCl pH 7.5). Lysozyme was added at a final concentration of 1 mg/mL (w/v), and samples were incubated at 37 °C for 1 h. Then, sodium dodecyl sulphate (SDS) and proteinase K were added, respectively, at a final concentration of 1% (v/v) and 0.5 mg/mL (w/v) and samples were incubated at 55 °C for 2 h in a water bath and periodically stirred. Total nucleic acids were extracted by phenol:chloroform:isoamylic alcohol (25:24:1 [v/v/v]) method according to standard procedures [107] and RNase A (final concentration 15 μg/mL) was used to remove contaminant RNA. After the extraction, high-molecular weight DNA was used as template in polymerase chain reactions (PCRs) to amplify the repetitive extragenic palindromic (REP) or BOX regions [108] and the partial length of the 16S rRNA encoding-gene.

For culture-independent analysis, the total intestine from male and female individuals was removed as described above and homogenized in a sterile tube containing glass beads (0.55 mm diameter) and 0.5 mL SET buffer for 15 min using a sterile pestle. Total DNA was then extracted using standard phenol-chloroform and ethanol precipitation method [107].

### BOX-PCR genomic fingerprinting

BOX-PCR genomic fingerprinting was done on all isolates as previously described [109] using the BOXA1-R primer (5’-CTACGGCAAGGCGACGCTGACG-3’). PCR products were separated on a 1% (w/v) agarose gel in 1× TBE buffer [107]. This analysis led us to identify 16 different genomic patterns (10 from female and 6 from male individuals). In order to better characterize these 16 gut isolates, having a different BOX profile, molecular identification was performed.

### 16S rRNA gene sequencing and phylogenetic analysis

Almost the entire 16S rRNA gene (from nucleotide 20 to nucleotide 1488 of the corresponding *E. coli* sequence) was amplified and sequenced by using the primer pairs 16SE20-42-F/16SEB683-R (corresponding to *E. coli* positions 20 to 683) [109, 110], Com1-F/Com2-R (corresponding to *E. coli* positions 519 to 926) [111], and 16SEB785-F/16SEB1488-R (corresponding to *E. coli* positions 785 to 1488) [109, 110]. These primer pairs amplified concatenated (and partially overlapping) DNA regions. PCR products were separated by agarose gel in 1× TAE buffer (40 mM Tris-acetate, 1 mM EDTA, pH 8.0), recovered by using the Qiaex II Gel extraction kit (Qiagen) and sequenced by using the same primers pair utilized for the respective amplifications by MWG Biotech Customer Sequencing Service (Germany). The sequences of bacterial isolates were compared with those of their closely related reference strains present in EzTaxon-e server [112]. Multiple sequence alignments between each pair of sequences were performed with ClustalW program at the Kyoto University Bioinformatic Center (http://www.genome.jp/tools/clustalw/) as previously described [113]. Phylogenetic trees were constructed using the SeaView software [114] according to the neighbour-joining (NJ) [115], and Kimura’s two-parameter algorithm [116]. Tree robustness was determined by bootstrap analysis based on 1,000 resamplings of data [117].

### Limited culture-independent analysis of gut bacteria

16S rRNA gene sequences from gut communities of 3 male and 3 female individuals (biological triplicates) were separately amplified using bacterial broad-range primer pairs Com1-F and 16SEB1488-R (amplicon length, 969 bp) in standard PCR. The relative amounts of amplifiable Firmicutes, α- and γ-Proteobacteria 16S rRNA gene sequences was determined by semi-quantitative real-time PCR using phylum/subphylum specific primer pairs.

Primer design was based on a representative phylogenetic dataset derived from the quality-checked and aligned sequences of the SILVA rRNA database project [118] (http://www.arb-silva.de). Primer sequences and amplicon lengths are reported below.

For semi-quantitative real-time PCR, each reaction was run on a SmartCycler system (Cepheid) with SsoAdvanced Universal SYBR Green Supermix (BIO-RAD) and the following primer pairs: Firm934-F/Firm1060-R (specific for Firmicutes 16S rRNA, amplicon length 126 bp) [119], Gamma877-F/Gamma1066-R (specific for γ-Proteobacteria 16S rRNA, amplicon length 189 bp) [119], and ADF681-F/ADF1362-R (specific for α-Proteobacteria 16S rRNA, amplicon length 681 bp) [120]. Com1-F/16SEB1488-R primer pair was used as a control for normalization.

Real-time PCR samples were run under these conditions: 3 min at 94 °C, 30 s at 94 °C, 30 s at 55 °C, 30 s at 72 °C for 35 cycles. Differences in cycle threshold (ΔCt) values between samples from males and females with phylum/subphylum-specific primers were normalized to differences in ΔCt with control primers.

### Microbiological assays

In order to determine whether any of the isolated gut bacterial strain could inhibit the growth of the others, microbiological assays were performed as follows. The bacterial strain that was assayed to detect antibiotic activity (referred to as “assayed bacterium” in Table 3) was grown until confluence on agar plates at 30 °C for 24 h. After growth, by using a sterile metallic cylinder (diameter 1 cm), agar disks with assayed bacteria were removed and positioned onto solid agar media (above mentioned) containing a suspension of the bacterium used to detect antibiotic activity (referred to as “tester bacterium” in Table 3) at a final concentration of 0.03 OD_600 nm_/mL. All gut bacteria from males and females were both crossed as “assayed” and “tester” microorganisms in various combinations. In addition, the following reference strains were used as tester microorganisms: *E. coli* strain FB8 [62], *S. enterica* subsp. *enterica* serovar Typhimurium strain LT2 ATCC 700720^T^ [63], *S. aureus* strain SA-1 [59], *M. luteus* strain ML-1 [60] and *S. ambofaciens* strain ATCC 23877 [61]. The rifamycin B producer *Amycolatopsis mediterranei* S699 was used as an antibiotic-producing control strain [121]. The following solid media were used: LB for the cultivation of all gut bacteria, *E. coli* strain FB8 and *S. enterica* subsp. *enterica* serovar Typhimurium strain LT2; NA for *S. aureus* strain SA-1 and *M. luteus* strain ML-1; HT for *S. ambofaciens* strain ATCC 23877; GYM for *A. mediterranei* S699. Each plate was incubated for 24 h at a different temperature depending of the tester bacterial strain utilized: 28 °C for *S. ambofaciens* strain ATCC 23877, 30 °C for all gut bacteria, 37 °C for *E. coli* strain FB8, *S*. Typhimurium strain LT2, *S. aureus* strain SA-1 and *M. luteus* strain ML-1. After this incubation time, a zone of growth inhibition can be seen around agar disk demonstrating the antibacterial activity of the assayed strain on tester microorganism. The diameters of the zones of growth inhibition were measured after subtracting the 1 cm-diameter of the agar disk containing the assayed bacterium, and the mean ± SD was calculated based on three independent experiments.

### Growth of gut bacteria on chemically defined media

We evaluated the ability of gut bacteria to grow in the chemically defined SRM, SRM-0 (without glucose) or SRM-oil media by replacing glucose with DMSO-dissolved *M. aquatica* essential oil (0.002%:0.25% [v:v]). Preliminary, the eventual side effect of dimethylsulfoxide (DMSO) on bacterial growth/survival was tested as previously described by Del Giudice et al. [100] with some modification. Briefly, log-phase bacterial cells grown without shaking at 30 °C were inoculated in 6 mL of SRM medium at a final concentration of about 1 × 10^5^ cells/mL. DMSO was added to reach the desired final concentration and the mixture was incubated at 30 °C under rotary shaking. 0.1 mL of the culture samples were collected at different intervals of time (24, 30 and 36 h), diluted appropriately (1:10, 1:100, 1:1000 in SRM) and plated on SRM agar in order to determinate the bacterial titers. To evaluate the effect of *M. aquatica* essential oil on bacterial growth, the essential oil was dissolved in 0.25% (v/v) DMSO, a suitable concentration with no inhibitory effect on the bacterial growth. No strain was able to grow when DMSO replaced glucose without essential oil.

### Growth of gut bacteria in the presence of *M. aquatica* essential oil

We also tested the capability of the bacterial isolates to grow both individually and in community by using the *M. aquatica* essential oil as sole carbon (and energy) source and biotransform it. Bacterial strains were grown individually until to middle logarithmic phase in SRM medium at 30 °C with rotary shaking. After this incubation time, cells were centrifuged, washed twice, re-suspended in SRM-0 medium (without glucose) and plated at appropriate dilutions (1:10, 1:100, 1:1000 in SRM) on solid SRM medium supplemented with a *M. aquatica* essential oil:DMSO emulsion (0.002%:0.25%,[v/v]). Then, the microorganisms that exhibited growth on solid SRM-oil medium, were individually inoculated into liquid SRM-oil medium until middle logarithmic phase at 30 °C for 48 h with rotary shaking in order to reach a mean value of 1.0 OD_600 nm_/mL. Finally, a 0.1 mL aliquot of each previous individual growth was taken and used to inoculate two bacterial communities into liquid SRM-oil medium. One community contained gut isolates from females (CHF-B4, CHF-B16, CHF-B17, CHF-B26, CHF-B37, CHF-G5, CHF-G14, CHF-PG1, CHF-PG3, CHF-PG4) and the other one containing those isolated from males (CHM-L11, CHM-L21, CHM-L22, CHM-N25, CHM-N28, CHM-N31). The two communities were incubated for about 40 h at 30 °C. After this period, bacterial cells were discarded and supernatant was further purified through Millipore filter (0.22 μm) before extraction (see below).

### SBSE-GC-MS profiling of bacterial metabolic transformations

Strains were grown in SRM medium to late logarithmic growth (about 30 h) at 28 °C with shaking. Bacteria were centrifuged, washed twice and re-suspended in SRM-0 at OD_600_ of 0.2 and incubated in 2 ml SRM-0 medium supplemented with both 8.0 mM L-glutamic acid, 20 μg/ml DMSO-dissolved *M. aquatica* essential oil for 40 h at 28 °C. Then bacterial cells were spin down and supernatant was further purified by Millipore filter (pore size 0.22 μm) before extraction. Compounds biotransformed by bacteria were extracted by using SBSE by direct immersion and stirring of SBSE into the supernatants as previously described [100].

### 16S rRNA GenBank Accession Numbers

The 16S rDNA nucleotide sequences of the 16 bacterial isolates were deposited at GenBank with the following accession numbers: *Serratia* sp. CHF-B4 (KP325087), *Serratia* sp. CHF-B16 (KP325088), *Serratia* sp. CHF-B17 (KP325089), *Serratia* sp. CHF-B26 (KP325090), *Serratia* sp. CHF-B37 (KP325091), *Serratia* sp. CHF-G5 (KP325092), *Pantoea* sp. CHF-G14 (KP325093), *Pseudomonas* sp. CHF-PG1 (KP325094), *Pseudomonas* sp. CHF-PG3 (KP325095), *Sphingomonas* sp. CHF-PG4 (KP325096), *Bacillus* sp. CHM-L11 (KP325097), *Bacillus* sp. CHM-L21 (KP325098), *Bacillus* sp. CHM-L22 (KP325099), *Pseudomonas* sp. CHM-N25 (KP325100), *Serratia* sp. CHM-N28 (KP325101), *Pseudomonas* sp. CHM-N31 (KP325102).

### Statistical analysis

In general all experiments were repeated at least three times. For chemical analyses, the experiments were repeated three times (biological replicates) with at least 15 plants and 30–50 insects for each experiment. Three technical replicates were run for each biological replicate. Fold change data are expressed as mean values, and standard deviations were generally less than 10% in all determinations. Analysis of variance (ANOVA) and the Tukey test were used to assess difference between samples.

## Additional files

Additional file 1: Figure S1. BOX-PCR genomic fingerprinting of the gut bacterial isolates from A) females and B) males of *C. herbacea* using BOXA1-R primer. M, 1 Kb DNA Ladder Invitrogen; 1, CHF-B26; 2, CHF-G5; 3, CHF-B37; 4, CHF-PG1; 5, CHF-PG3; 6, CHF-B4; 7, CHF-PG4; 8, CHF-G14; 9, CHFB16; 10, CHF-B17; 11, CHM-L11; 12, CHM-L21; 13, CHM-L22; 14, CHM-N25; 15, CHM-N28; 16, CHM-N31. **Figure S2.** Examples of antibacterial activities (demonstrated by halo of growth inhibition around assayed microorganism). **A**, Assayed bacteria: CHF-B4, CHF-B16, CHF-B17, CHF-B26, CHFB37, CHF-G5; Tester bacterium: CHM-L11. **B**, Assayed bacteria: CHF-B4, CHF-B16, CHF-B17, CHF-B26, CHF-B37, CHF-G5; Tester bacterium: CHM-L11. **C**, Assayed bacterium: *A. mediterranei* S699; Tester bacteria (from left to right): CHM-L11, CHM-L21 and CHM-L22. For details refer to the Methods section. (PDF 1546 kb)

Additional file 2: File S1. Relative abundance of Firmicutes, α- and γ-Proteobacteria 16S rRNA gene sequences in *C. herbacea* male and female guts as determined by real-time PCR using phylum/subphylum specific primer pairs. The file reports raw data (yellow background), data analysis and statistical processing from real-time PCR experiments. Samples #1, #2 and #3 represent biological triplicates. (XLSX 47 kb)

## Abbreviations

ANOVA: Analysis of variance
CFU: Colony-forming units
DMSO: Dimethylsulfoxide
DVB/CAR/PDMS: Divinylbenzene/Carboxen/Polydimethylsiloxane
EDTA: Ethylenediaminetetraacetic Acid
GC-MS: Mono-dimensional gas chromatography–mass spectrometry
GCxGC-MS: Two-dimensional gas chromatography–mass spectrometry
GYM: Glucose Yeast Malt
HS-SPME: Headspace solid-phase microextraction
HT: Hickey-Tresner
ISTD: Internal Standard
LB: Luria-Bertani
MVOCs: Microbial volatile organic compounds
NA: Nutrient Agar
NJ: Neighbour-joining
REP: Repetitive extragenic palindromic
SBSE: Stir Bar Sorptive Extraction
SD: Standard deviation
SDS: Sodium dodecyl sulphate
SET: Sodium EDTA Tris–HCl
TAE: Tris Acetic Acid EDTA
TBE: Tris Boric Acid EDTA
YEPD: Yeast Extract Peptone Dextrose
ΔCt: Cycle threshold
