# Supplementary material for: Cultivable gut bacteria provide a pathway for adaptation of Chrysolina herbacea to Mentha aquatica volatiles
Source: BMC Plant Biol. 2017 Mar 2;17:30. doi: 10.1186/s12870-017-0986-6 (PMC5333409; doi:10.1186/s12870-017-0986-6)
Supplement: Supplementary file 1 — BOX-PCR genomic fingerprinting of the gut bacterial isolates from A) females and B) males of C. herbacea using BOXA1-R primer. M, 1 Kb DNA Ladder Invitrogen; 1, CHF-B26; 2, CHF-G5; 3, CHF-B37; 4, CHF-PG1; 5, CHF-PG3; 6, CHF-B4; 7, CHF-PG4; 8, CHF-G14; 9, CHFB16; 10, CHF-B17; 11, CHM-L11; 12, CHM-L21; 13, CHM-L22; 14, CHM-N25; 15, CHM-N28; 16, CHM-N31. Figure S2. Examples of antibacterial activities (demonstrated by halo of growth inhibition around assayed microorganism). A, Assayed bacteria: CHF-B4, CHF-B16, CHF-B17, CHF-B26, CHFB37, CHF-G5; Tester bacterium: CHM-L11. B, Assayed bacteria: CHF-B4, CHF-B16, CHF-B17, CHF-B26, CHF-B37, CHF-G5; Tester bacterium: CHM-L11. C, Assayed bacterium: A. mediterranei S699; Tester bacteria (from left to right): CHM-L11, CHM-L21 and CHM-L22. For details refer to the Methods section. (PDF 1546 kb) [file 12870_2017_986_MOESM1_ESM.pdf]

Supplementary Figure

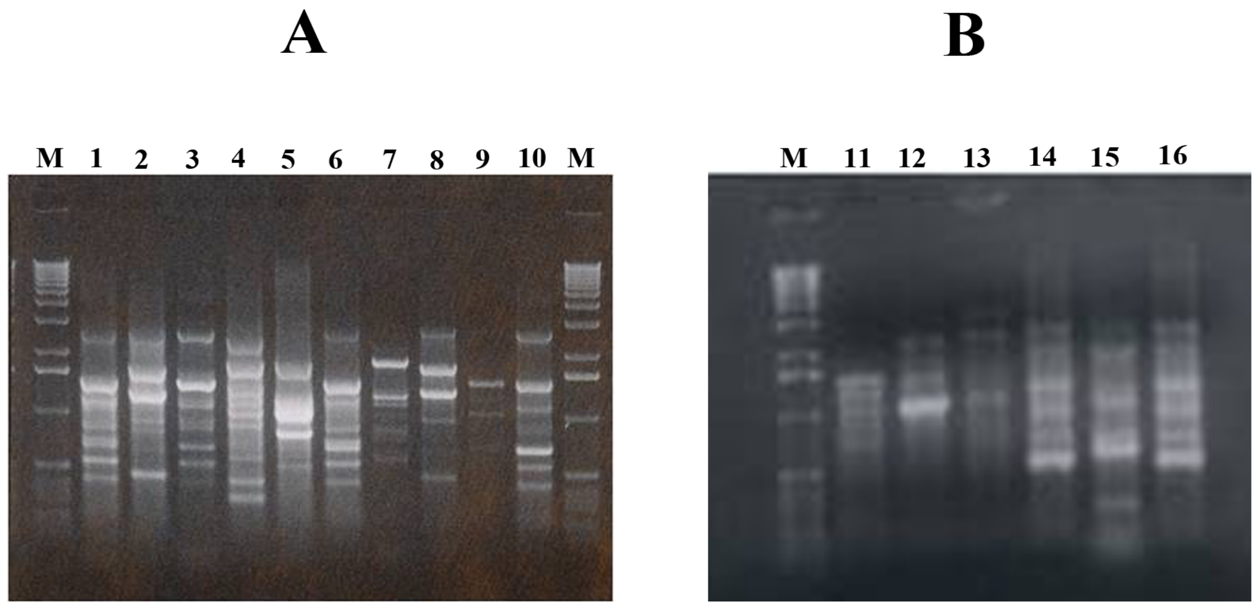

Fig. S1 BOX-PCR genomic fingerprinting of the gut bacterial isolates from A) females and B) males of *C. herbacea* using BOXA1-R primer. M, 1 Kb DNA Ladder Invitrogen; 1, CHF-B26; 2, CHF-G5; 3, CHF-B37; 4, CHF-PG1; 5, CHF-PG3; 6, CHF-B4; 7, CHF-PG4; 8, CHF-G14; 9, CHF-B16; 10, CHF-B17; 11, CHM-L11; 12, CHM-L21; 13, CHM-L22; 14, CHM-N25; 15, CHM-N28; 16, CHM-N31.

A

B

C

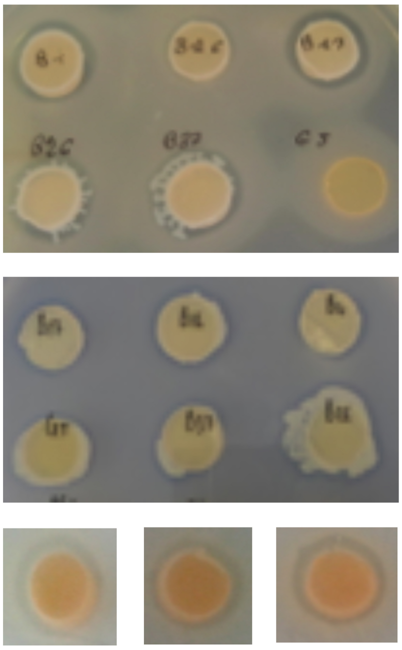

Fig. S2 Examples of antibacterial activities (demonstrated by halo of growth inhibition around assayed microorganism). **A**, Assayed bacteria: CHF-B4, CHF-B16, CHF-B17, CHF-B26, CHF-B37, CHF-G5; Tester bacterium: CHM-L11. **B,** Assayed bacteria: CHF-B4, CHF-B16, CHF-B17, CHF-B26, CHF-B37, CHF-G5; Tester bacterium: CHM-L11. **C,** Assayed bacterium: *A. mediterranei* S699; Tester bacteria (from left to right): CHM-L11, CHM-L21 and CHM-L22. For details refer to the Methods section.
